# Neuroglial CB1 receptors modulate hippocampal processes in a sex-dependent manner

**DOI:** 10.1186/s13293-026-00886-w

**Published:** 2026-03-19

**Authors:** Jon Egaña-Huguet, Lucía Sangroniz-Beltrán, Andrés M. Baraibar, Pablo Reyes-Velásquez, Nicolas F. Landgraf, Paula Torres-Maldonado, Cathaysa Rodríguez-Cedrés, Francisca Julio-Kalajzic, Joaquín Piriz, Almudena Ramos, Pedro Grandes, Giovanni Marsicano, Susana Mato, María Ceprián, Edgar Soria-Gómez

**Affiliations:** 1 Department of Neurosciences, University of the Basque Country UPV/EHU, Leioa, 48940 Spain; 2 Achucarro Basque Center for Neuroscience, Leioa, 48940 Spain; 3 Neuroimmunology Unit, IIS Biobizkaia, Barakaldo, 48903 Spain; 4 U1215 Neurocentre Magendie, Endocannabinoïdes et neuroadaptation, Institut national de la santé et de la recherche médicale (INSERM), Bordeaux, 33000 France; 5 Ikerbasque, Basque Foundation for Science, Bilbao, 48009 Spain

**Keywords:** CB1 receptors, Hippocampus, Astrocytes, Neurons, Navigation, Memory, Sex differences

## Abstract

**Background:**

Navigation and memory are hippocampal functions that are essential for survival. One of the key modulators of hippocampal activity is the endocannabinoid system through the cannabinoid receptor type 1 (CB1). CB1 is widely expressed in various types of hippocampal cells. While it is known that CB1 participates in memory processes, its specific roles in different cell types and differences between sexes remain unclear.

**Methods:**

This study investigates the cell- and sex-specific modulation of navigation and memory by CB1 receptors. To this end, we selectively deleted CB1 receptors from hippocampal neurons or astrocytes in adult male and female mice. We then assessed its effect on a comprehensive range of behaviors, including innate emotional responses, memory, navigation, and nesting.

**Results:**

Deletion of CB1 from hippocampal principal cells or astrocytes produced significant changes in innate emotional behavior and novel object recognition in both sexes. Conversely, CB1 receptor deletion from all neurons produced deficits in spatial navigation exclusively in males. Interestingly, synaptic plasticity was impaired in both male and female mice lacking CB1 receptors from GFAP-positive cells. Nest-building behavior was only affected in males carrying the CB1 deletion in CAMK-positive cells.

**Conclusion:**

In conclusion, our findings show that neuronal CB1 receptors are critical for spatial navigation in males, while astrocytic CB1 receptors play a key role in memory processes in both males and females.

**Supplementary Information:**

The online version contains supplementary material available at 10.1186/s13293-026-00886-w.

## Abstract (plain language summary)

Memory and navigation are crucial for animals to survive. They need them to locate food and reproductive sources, as well as to escape and to avoid potentially dangerous environments. Such needs could vary in a sex-dependent manner. Our work aims to study the biological mechanisms underlying these processes. Different brain regions work together to achieve this goal. In particular, the hippocampus plays a key role. Hippocampal function is regulated by the orchestrated activity of different cell types, including neurons and glial cells, which are tightly regulated by modulatory systems. In this study, we use the cannabinoid receptor type-1 (CB1) as a molecular tool to decipher the specific contributions of hippocampal neurons and glia to memory and navigation processes in male and female mice. Our results showed that glial activity is necessary for general learning, recognition memory, and synaptic plasticity in both sexes. Interestingly, only male mice seem to rely exclusively on hippocampal neuronal activity for spatial navigation and early phases of synaptic plasticity. In summary, we found that the CB1 receptor modulates hippocampal functions in a sex-dependent manner, with clear cell-type-specific effects.

**Supplementary Information:**

The online version contains supplementary material available at 10.1186/s13293-026-00886-w.

## Highlights

- Neuronal CB1 receptors in the hippocampus are crucial for spatial navigation in males, but not females.
- Astroglial CB1 receptors in the hippocampus modulate learning and memory processes in both sexes.
- Synaptic plasticity is impaired in male and female mice lacking the CB1 receptor in hippocampal astrocytes.
- Neuronal CB1 deletion in the hippocampus only affects the early phase of synaptic plasticity in males but not females.

**Supplementary Information:**

The online version contains supplementary material available at 10.1186/s13293-026-00886-w.

## Background

Navigating a landscape is essential for species survival. Animals utilize environmental cues (e.g., visual signals, odors) and previously learned information to achieve specific goals, such as finding shelter or avoiding danger. The strategies employed to perform these behaviors stem from a balance between following spatial cues, flexibility in their interpretation, and efficiently executing experience-guided tasks. In this manner, internal maps are created and can later be utilized to enhance behavioral efficacy [1–3]. In a novel environment, exploratory behavior may seem disorganized. However, as the environment becomes familiar, vector-based navigation (allocentric strategy) and egocentric strategies typically emerge [4–7]. Nonetheless, the process of selecting different strategies for specific environments remains unclear.

The hippocampus (HC) [4–6], along with other brain regions [7, 8], modulates specific strategies and memory formation. Although the hippocampal formation operates as a unified entity in memory processes, the areas along the dorsoventral axis serve distinct roles. The projections received by the HC from cortical or subcortical regions also differ between the dorsal and ventral areas [9]. The dorsal part primarily processes previously acquired spatial information, while the ventral part plays a more significant role in forming new environmental layouts [10].

Different cell types are identified as key components of the navigation process. From place cells in the CA1 region of the hippocampus, which form neural ensembles activated when the animal visits a specific location, to grid cells in the entorhinal cortex, which integrate information about position, direction, and distance [11]. Furthermore, glial cells, particularly astrocytes, also play a fundamental role in acquiring and utilizing environmental information [12, 13]. Accordingly, several neuromodulatory systems regulate these functions, with the endocannabinoid system being one of the most studied [14–16]. In particular, cannabinoid receptor type-1 (CB1) is widely expressed in hippocampal cells, including neurons and glia [17, 18]. It is also found in subcellular domains, such as mitochondrial membranes [19–21]. At the functional level, neuroglial CB1 receptors in the hippocampus are essential for synaptic plasticity and memory formation [22–24].

Most of these studies were conducted on male rodents. However, recent research indicates that the role of astrocytes in memory recall and navigation varies between male and female mice [25]. Stress has also been found to affect spatial learning differently in both sexes, impairing learning only in males [26–28]. This sexual dimorphism is also evident in endocannabinoid levels [29, 30] and CB1 distribution [31, 32]. In this context, little is known about the role of hippocampal CB1s in females, where their involvement in hippocampal-dependent processes appears to differ from that in males.

In this study, we aim to investigate the cell-specific and sex-dependent role of CB1 signalling in hippocampus-dependent behavioral processes. To achieve this, we specifically deleted neuronal or astrocytic CB1 receptors in the hippocampus of adult male and female mice and assessed their impact on innate emotional responses, hippocampus-dependent memory and navigation, and synaptic plasticity.

Our tests show that innate emotional behavior and object recognition are disrupted when CB1 is deleted from hippocampal astrocytes or principal cells in females and males. Conversely, neuronal CB1 is essential for the development of spatial navigation strategies only in male mice. However, loss of CB1 from astrocytes hinders learning and memory recall in both males and females. Finally, astrocytic CB1 deletion produced a robust decrease in synaptic plasticity in both sexes. A similar effect was observed in male, but not female, mice lacking neuronal CB1 receptors.

## Methods

### Animal models

All experimental procedures were approved by the Animal Research Ethical Committee of the University of País Vasco (M20/2019/199 // M30/2019/301). *CB*_*1*_-flox animals were a generous gift from Prof. Manuel Guzmán (Complutense University of Madrid). *CB*_*1*_-KO mice were locally produced at the University of País Vasco Animal Facility. Animals were maintained in individual cages under standard conditions with water and food *ad libitum*.

### Viral vectors

Adenoviral vectors (ssAAV) were purchased from the Viral Vector Facility, VVF(University of Zurich). The vectors ssAAV-9/2-hSyn1-mCherry_iCre-WPRE-hGHp(A), ssAAV-9/2-hGFAP-mCherry_iCre-WPRE-hGHp(A) and ssAAV-9/2-mCaMKIla-mCherry 2 A iCre-WPRE-SV40p(A) were used to selectively deliver Cre expression to neurons, astrocytes and principal glutamatergic neurons, respectively. ssAAV-9/2-hSyn1-chl-mCherry-WPRE-SV40p(A), ssAAV-9/2-hGFAP-mCherry-WPRE-hGHp(A) and ssAAV-1/2-mCaMKIIα-mCherry-WPRE-hGHp(A) were used as control (Table 1).

**Table 1:** ssAAV Viral vectors used in the different experiments

ssAAV Viral Vector	Group Name
ssAAV-9/2-hSyn1-mCherry-iCre-WPRE-hGHp(A)	HC-(SYN)*CB*_*1*_-KO
ssAAV-9/2-hGFAP-mCherry-iCre-WPRE-hGHp(A)	HC-(GFAP)*CB*_*1*_-KO
ssAAV-9/2-mCaMKIla-mCherry-2 A-iCre-WPRE-SV40p(A)	HC-(CAMKII)*CB*_*1*_-KO
ssAAV-9/2-hSyn1-chl-mCherry-WPRE-SV40p(A)	CTL
ssAAV-9/2-hGFAP-mCherry-WPRE-hGHp(A)	CTL
ssAAV-1/2-mCaMKIIα-mCherry-WPRE-hGHp(A)	CTL

### Surgery and viral vector administration

### Behavioral tests

**Fig. 1:**
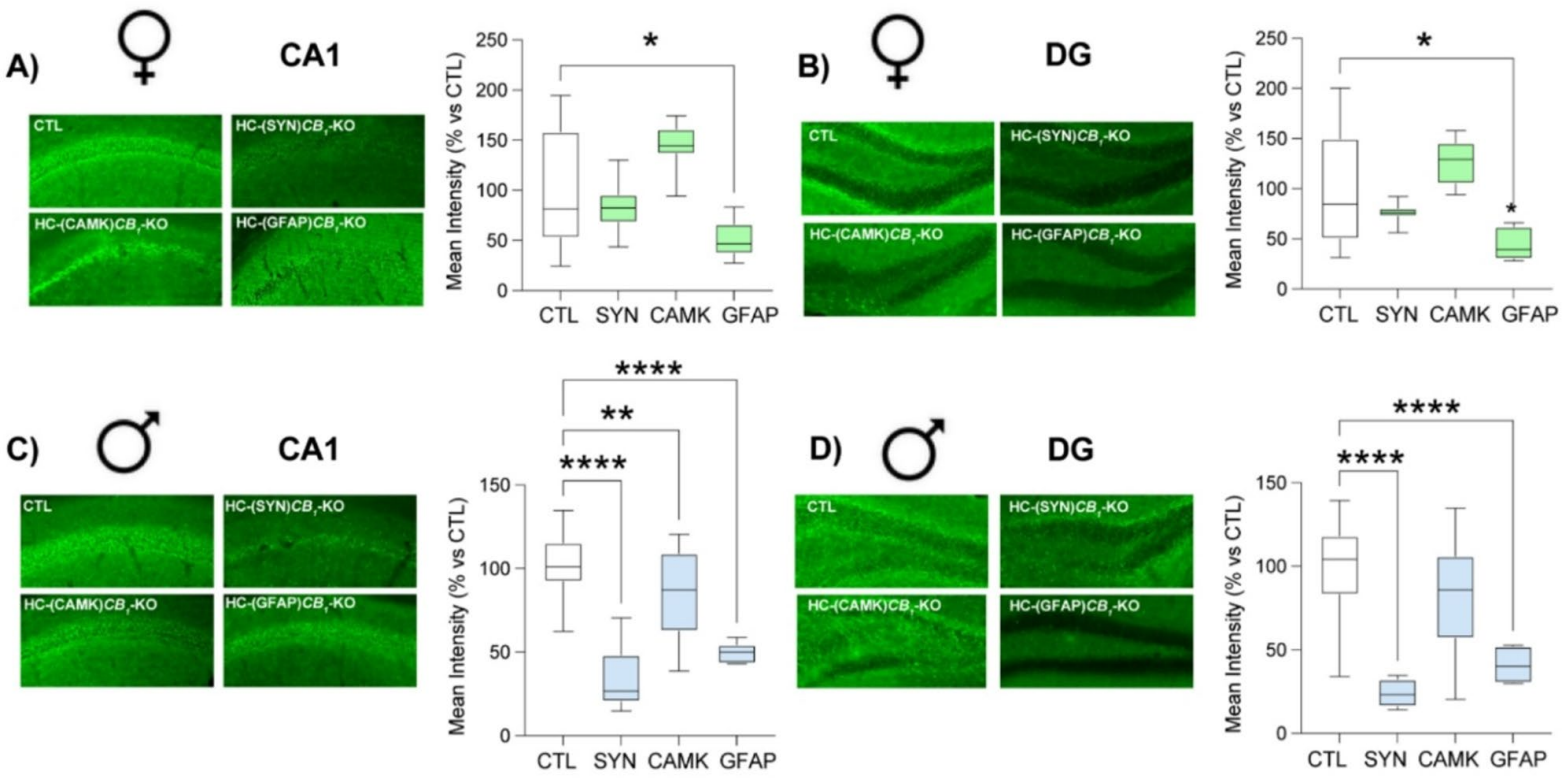
Hippocampal CB1 deletion. Quantification of CB1 immunofluorescence in the CA1 **(A**, **C)** and DG **(B**, **D)** regions of the hippocampus five weeks after the injection of AAV expressing Cre recombinase under different cell-type–specific promoters: the pan-neuronal promoter SYN, the glutamatergic promoter CAMKII, and the astrocytic promoter GFAP, in both female **(A**, **B)** and male **(C**, **D)** mice. Statistical analysis by One-Way ANOVA test followed by Holm-Sidak’s multiple comparison tests; * *p* < 0.05, ** *p* < 0.01, ****p < 0.0001 vs. CTL. See Table S1 for detailed statistics

To delete CB1 in the HC, two-month-old male and female *CB*_*1*_-flox mice were bilaterally injected in the dorsal hippocampus with the AAVs using the RWD stereotaxic system (RWD; Guangdong, China). The animals were anaesthetized with isoflurane at 5% for induction, then maintained at 1% during surgery. Bilateral injections of 250 nl were carried out at the coordinates − 2 AP, ± 1.5 ML, and − 2 DV, using the Nanoject III system (Drummond Scientific; Broomall, PA, USA). Breathing and body temperature were monitored throughout the procedure. Lidocaine was topically administered at the beginning, and carprofen (4 mg/kg) was subcutaneously injected at the end of the surgery. The animals were individually caged with humidified food and observed for three days. Four to five weeks after AAVs hippocampal injection, animals were sacrificed, and brains were processed to verify the site of injection using a fluorescent co-immunostaining for the virus fluorescent protein (mCherry) together with neuronal (NeuN) or astrocytic (GFAP) markers (Fig. S1) and the deletion of CB1 (Fig. 1).

### Behavioral tests

Animals were allowed to recover for 4 weeks after surgery to ensure expression of the adenoviral vector and to assess its effect on disrupting *de novo* receptor synthesis (Fig. 1 and Fig. S1). Behavioral tests were conducted during the light phase, once per day. Animals were allowed to acclimate for 30 min in the room before the start of the experiments. Tests were recorded and analyzed using the ANY-maze system (ANY-maze; Dublin, Ireland). The light intensity in the behavioral room was maintained at 200 lx, except in the light-dark box, where we used 1200 lx in the light compartment.

### Innate emotional responses

*Open Field*: Animals were positioned in the center of a dimly lit open-field arena (40 × 40 × 45 cm) and allowed to explore freely for 5 minutes. A square area of 6 × 6 cm, located 6 cm from the walls, was designated as the “center zone,” while the remainder was defined as the “safe zone”. Time and the number of entries in the “center zone” were analyzed. *Elevated Plus Maz*e: Mice were placed in the center of an Elevated Plus Maze (4 arms measuring 30 × 5 cm; 2 open and 2 closed) at a height of 49 cm and allowed to explore the maze freely for 5 minutes. Time spent and the number of entries in the open arms were quantified. *Light-Dark Box*: Mice were placed in a brightly illuminated arena (1200 lux) connected to a dark chamber (30 × 30 × 35 cm). Time spent and the number of entries into the illuminated area were analyzed.

### Memory and navigation

#### Novel object recognition test

The Novel Object Recognition Test (NORT) was conducted using an L maze (with external and internal L walls measuring 35 cm and 30 cm, respectively, and wall dimensions of 4.5 cm wide and 15 cm high) [33, 34]. All stages were recorded using an iDS camera paired with a uEYE software system. On the first day (habituation), mice were allowed to explore the maze for 9 min. On the second day (acquisition), two identical objects were placed at the end of each arm, and mice were given 9 min to explore them before returning to their home cage. The test took place 24 h later, with one of the objects replaced by a new, unfamiliar one. The animal was then allowed to explore both objects for an additional 9 min.

The EthoVision0 (Noldus) software was used to quantify the time spent exploring each object. The discrimination index (DI) was calculated as the difference between the time spent exploring the novel object (TN) and the familiar object (TF), divided by the total exploration time (TN + TF): DI = [TN-TF]/[TN + TF] [24]. All objects were previously assessed so that animals would not show any preference.

#### Barnes maze

To assess memory and navigation, animals were subjected to the Barnes Maze (BM). The BM is a circular platform of 150 cm in diameter, with 20 holes in the vicinity, 90 cm above the floor. Animals performed BM for 5 days, four trials per day. To complete each trial, they may use up to two minutes, with one resting minute in the home cage between tests. Meanwhile, the maze was cleaned. On the first day, animals are driven to the target hole before the trials begin. The test begins in the center of the maze, where mice are placed in an opaque plastic cylinder for 10 s before being released. Visual cues are located around the room to help guide the use of spatial cues to resolve the maze.

The time to reach the target hole, the number of errors, and the strategy used were analyzed. Any hole inspected before reaching the target hole is considered an error. Solving strategies were classified as previously described [5, 35]. Briefly, when the animal reaches the hole without following any pattern or missing two consecutive holes, it is considered Random. A Serial strategy is defined when the animal reaches the hole after exploring more than one hole consecutively, with only one hole missing between them. Finally, a strategy is considered Spatial when the animal goes directly to the target or to two nearby holes. If a mouse does not get to the target hole within 4 min, it will be gently guided to it. After each trial, mice were left in the escape box for 1 min before being moved to the home cage.

### Ex-vivo electrophysiology

We collected acute coronal hippocampal slices (350 μm thick) from *CB*_*1*_-flox mice (males and females) carrying a specific CB1 deletion in hippocampal neurons (SYN), glutamatergic neurons (CAMKII), or astrocytes (GFAP). Slices were kept in ice-cold artificial CSF (ACSF) containing the following (in mM): 124 NaCl, 5 KCl, 1.25 NaH_2_PO_4_, 2 MgSO_4_, 26 NaHCO_3_, 2 CaCl_2_, and 10 glucose gassed with 95% O_2_/5% CO_2_, pH 7.3–7.4. Slices were incubated in ACSF at room temperature for at least 1 h before use, then transferred to an immersion recording chamber, superfused at 2 mL/min with gassed ACSF, and visualized under an Olympus BX51WI microscope (Olympus Optical, Japan). Field excitatory postsynaptic potentials (fEPSPs) were evoked in the CA1 stratum radiatum by stimulating Schaffer collaterals (SCs) with theta capillaries (2–5 μm tip) filled with ACSF and recorded with ACSF-filled glass pipettes (< 5 MΩ). Electrical pulses were supplied by a stimulus isolation unit (ISO-Flex, A.M.P.I., Jerusalem, Israel). Signals were fed to a Pentium-based PC through a DigiData 1550 interface board. Signals were filtered at 1 kHz and acquired at a 10 kHz sampling rate using a DigiData 1550 data acquisition system. pCLAMP 10.3 software (Molecular Devices, San Jose, CA) was used for stimulus generation, data display, acquisition, and storage. For baseline recording, the stimulation intensity was adjusted to obtain 40% of the maximum slope of the response, and inputs were stimulated (1 ms pulse duration) every 5 s. The slope of the fEPSPs was measured between 30 and 70% of the maximum. For LTP induction, a high-frequency stimulation (HFS) protocol (4 trains at 100 Hz for 1 s; 30 s intervals) was applied to the SCs. fEPSP slope was normalized to the 10 min of baseline recording. After LTP induction, fEPSPs were recorded for 60 min. The presence of LTP was determined by comparing the average fEPSP slope during the baseline with the mean values from the 10–30 min (early LTP) and 30–60 min (late LTP) post-induction periods.

### Tissue processing

Once behavioral tests were performed, mice were deeply anesthetized by an intraperitoneal administration of a mixture of Ketamine/Xylazine (80/10 mg/kg body weight). Right after, they were transcardially perfused with PBS (0.1 M, pH 7.4) for 1 min before being fixed with 4% formaldehyde solution for another 7 min with a peristaltic pump at 12 ml/min (Perimax 12; Spetec GmbH, Erding, Germany). Brains were extracted and post-fixed in a 4% formaldehyde for one week at 4 °C. For their storage, the fixative solution was diluted 1:10 and brains were kept at 4 °C until use.

#### Immunofluorescence

Stored brains were cut into 50 μm slices using a vibratome (Leica, VT1000S) and kept in PB 0.1 M + azyde 0.05%. To analyze the depletion of the CB1 receptor, slices from AP -1.7 to AP -2.3 were selected. Slices were washed with PB 0.1 M to clean the azyde and then blocked for 1 h at room temperature (RT) with blocking buffer (PB 0.1 M + donkey normal serum at 10% (S30M, Sigma-Aldrich; St. Louis, USA) + 0.3% triton X-100 (X100-5ML, Sigma-Aldrich; St. Louis, USA). Slices were then incubated with primary antibodies for 72 h at 4 °C. To verify the viral vector injection site, together with the co-localization in astrocytes, several antibodies were used: goat antibody against CB1R (anti-CB_1_R; 1:500; Frontier Institute co., ltd; CB1-Go-Af450; RRID: AB_2571591) mouse anti-Hexaribonucleotide Binding Protein-3 (α-NeuN; RRID_AB104224, Abcam; Cambridge, UK) at 1:1000; rabbit anti-Cherry monomer (α-mCherry, RRID_AB356482, Abcam; Cambridge, UK) at 1:500; mouse anti-glial fibrillary acidic protein (α-GFAP; RRID_AB257130, Abcam; Cambridge, UK) 1:1000. Slices were washed with PB 0.1 M before adding secondary antibodies for 3 h at RT. The secondary antibodies were either α-rabbit AlexaFluor 594 (RRID_AB2534016), α-mouse AlexaFluor 488 (RRID_AB141607). Finally, mouse brain slices were washed and mounted using Fluoroshield Mounting Medium with DAPI (F6057; Sigma-Adrich; St. Louis, USA). Microphotographs from hippocampi were taken using a Nikon TiU microscope with a Nikon Ds-Qi2 camera or in a Zeiss Apotome microscope attached to an Axiocam 506 mono y ER5c camera. CB1 receptor protein immunolabeling was then analyzed using ImageJ software [36], in which the staining intensity in each condition was compared by measuring the mean fluorescent intensity in the CA1 and dentate gyrus (DG) regions, after background staining was removed from each area.

### Nest building

Nests were collected every 2 weeks. Nest pictures were obtained using a black cardboard with a ruler and a camera (Luminex Panasonic, DMC-FZ200; Panasonic Spain, Cornellá de Llobregat, Spain). The percentage of remaining tissue relative to the intact full paper is calculated in FIJI to assess the degree of nesting complexity (adaptation of Deacon 2006) [37]. The images are transformed to 8-bit and the threshold is set to 125. The remaining tissue area is calculated and normalized to the original paper size.

### Experimental design and statistical analysis

For the statistical analysis, we used GraphPad 9 software (GraphPad Prism version 9.0.0 for Windows, GraphPad Software, Boston, Massachusetts USA, www.graphpad.com). First, data normality was assessed using the Kolmogorov-Smirnov test. For the innate emotional response, NORT, and nesting analysis, an ordinary One-Way ANOVA was applied, followed by a post-hoc Holm-Sidak’s multiple comparisons test. If the data failed the normality test, a Kruskal-Wallis nonparametric test was applied, followed by Dunn’s post hoc multiple-comparison test.

BM results were analyzed and compared as the daily mean for each trial. Additionally, the 5-day mean was used to compare genotypes based on latency, number of errors, and strategies. A two-way ANOVA was performed, followed by Sidak’s multiple comparisons test. Electrophysiology ex vivo was characterized by a two-tailed unpaired Student *t* test or One-Way ANOVA. When data did not meet normality assumptions, a Mann-Whitney or Kruskal-Wallis test was used. The results were considered significant when the p-value was equal to or lower than 0.05.

## Results

### Innate emotional responses

Five weeks after the injection, the animals underwent a series of behavioral tests (open field, elevated plus maze, and light-dark box) to assess their innate emotional responses to aversive stimuli, ranging from the least to the most stressful.

**Fig. 2:**
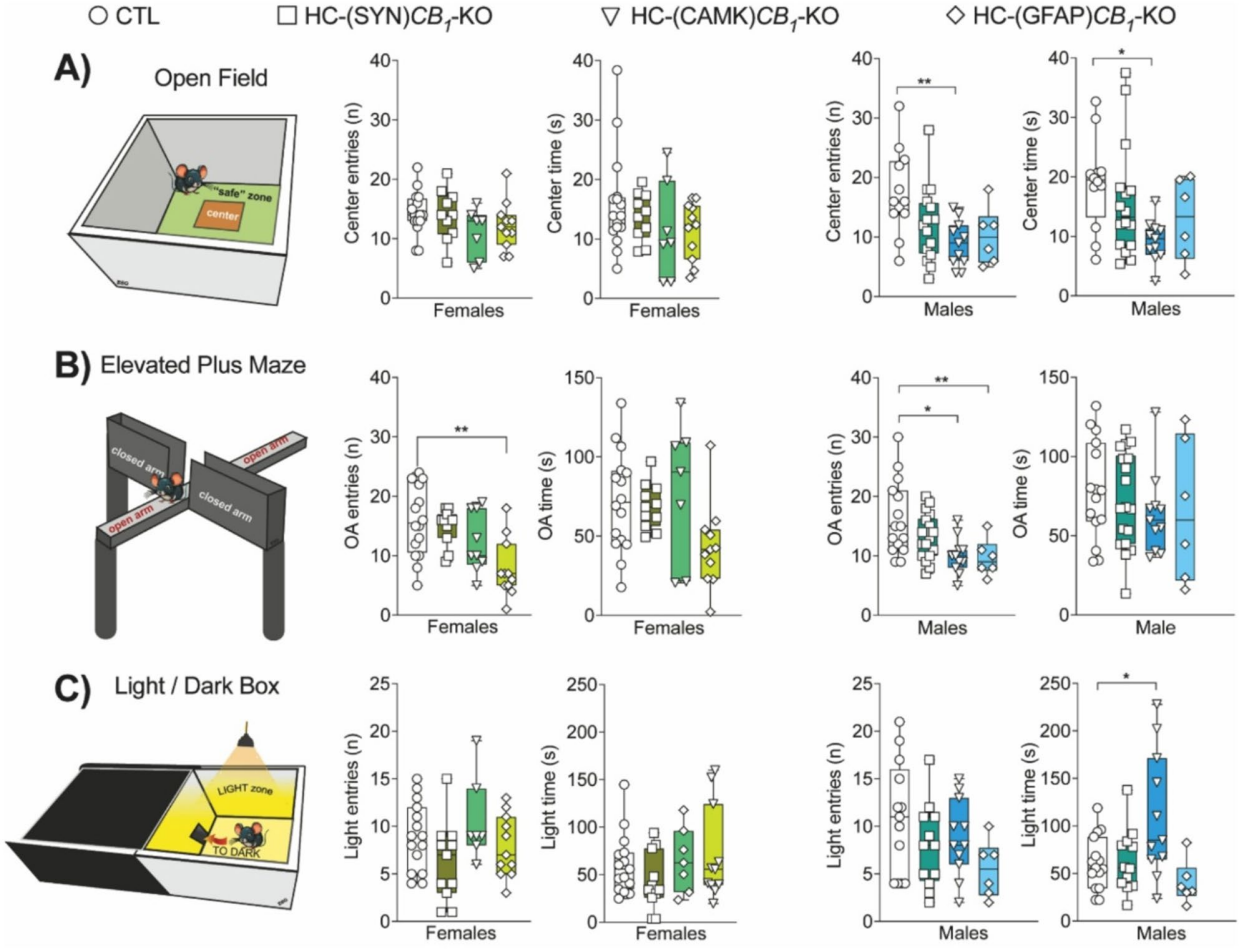
Innate emotional behavior. Differences in females *(green charts)* were only found in the number of entries to the open arms for the HC-(GFAP)*CB*_*1*_-KO **(B).** Male animals *(blue charts)* with CB1 deletion in CAMKII neurons showed an anxiogenic profile with lower entries and time in the center of the open field **(A)** and in the open arms entries (OA) of the elevated plus maze **(B)** and an increased time in the light zone of the light / dark box **(C)** compared with CTL animals. Data are presented as mean ± S.E.M. Statistical analysis by One-Way ANOVA test followed by Holm-Sidak’s multiple comparison tests; * *p* < 0.05, ** *p* < 0.01 vs. CTL. See Table S1 for detailed statistics

Receptor deletion had different effects on the genotypes and sexes assessed. Differences were mainly found in male mice lacking the CB1 receptor from neurons, and in females carrying the deletion in astrocytes. The clearest phenotype was observed when CB1 was specifically deleted from principal cells ((CAMK)*CB*_*1*_-KO) in the hippocampus of males. HC-(CAMK)*CB*_*1*_-KO male mice exhibited a significant decrease in the number of entries and the time spent in the center zone of the open field test (Fig. 2A) and entries in the open arms of the elevated plus maze (Fig. 2B), accompanied by an increase in the time spent in the light zone of the L-D box (Fig. 2C) compared to controls. Regarding astrocytic CB1 receptors, a significant decrease in the entries into the open arms was observed in HC-(GFAP)*CB*_*1*_-KO male and female mice compared with control animals (Fig. 2B). In female mice, CB1 deletion did not induce a clear emotional alteration, with the only differences observed in the elevated plus maze test for the HC-(GFAP)*CB*_*1*_-KO female genotype (Fig. 2B). Interestingly, full knockout mice (*CB*_*1*_ -KO) showed anxiety-like behavior profile for both males and females (Fig. S2), especially in the elevated plus maze (Fig.S2B).

Taken together, our results show that CB1 receptors present in CAMKII-positive principal neurons modulate innate emotional response in males but not in females. Conversely, astrocytic CB1 receptors seem to be more relevant in females, at least in the elevated plus maze.

### Learning and memory

**Fig. 3:**
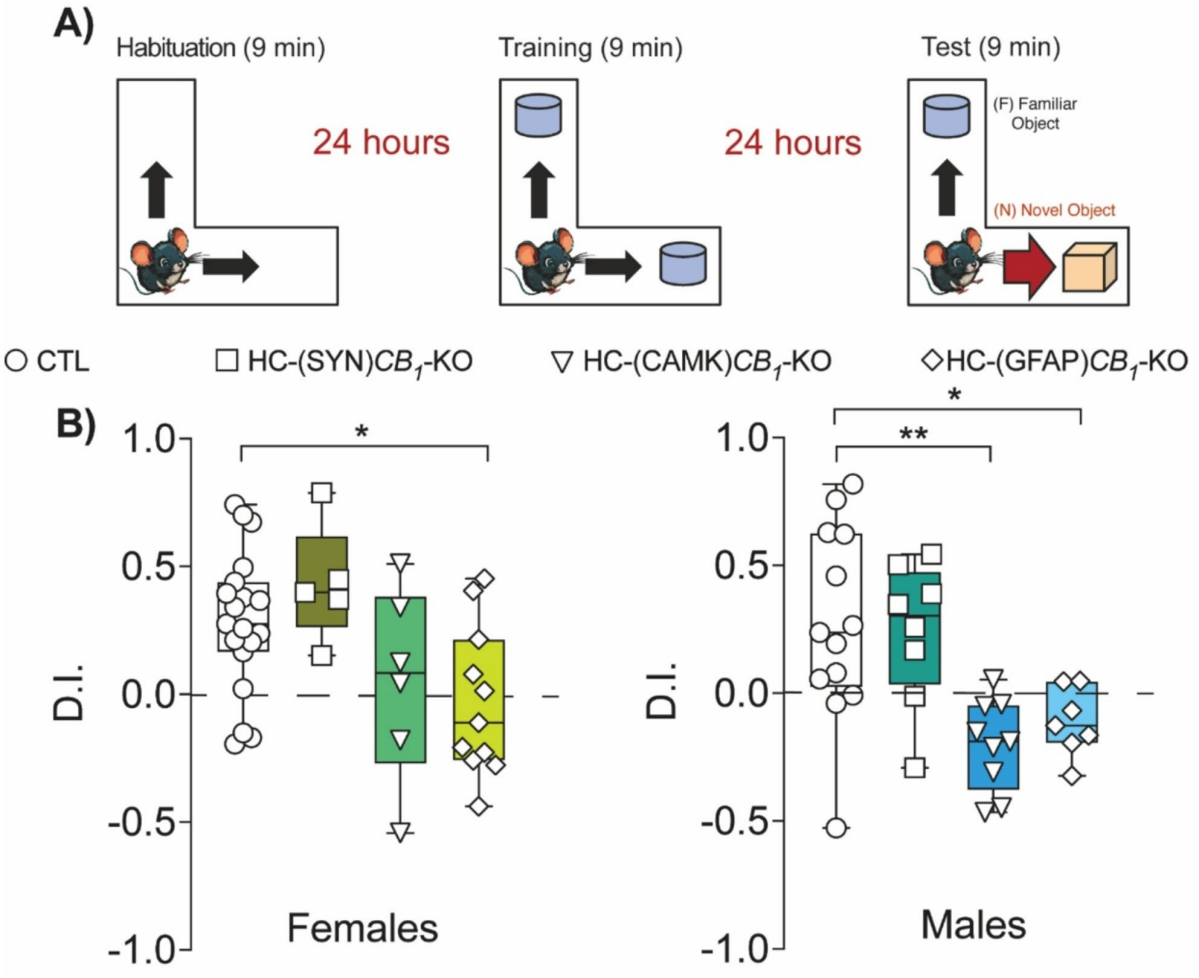
Novel object recognition. **(A)** Animals were trained with two identical objects for 9 min. 24 h later, the animals were re-exposed, and one of the objects was changed for a new one. HC-(GFAP)*CB*_*1*_-KO females had a lower D.I. than CTLs **(B**,** left panel)**. In males, CB1 deletion in principal neurons or astrocytes reduced D.I compared with CTLs **(B**,** right panel)**. Data are presented as mean ± S.E.M. Statistical analysis by One-Way ANOVA test followed by Holm-Sidak’s multiple comparison test. * *p* < 0.05, ** *p* < 0.01 vs. CTL. See Table S1 for detailed statistics

To address the effects of sex and CB1 cellular deletion on hippocampal-dependent memory processes, we used the novel object recognition and Barnes maze (BM) tests. To assess recognition memory, we performed the NOR test in the L maze (Fig. 3A). Astrocytic CB1 deletion impaired memory in both females and males, significantly reducing the D.I. (Fig. 3B). In male mice, a significant reduction was also observed when CB1 was deleted from CAMKII-positive cells (Fig. 3B). There was no significant impairment in *CB*_*1*_ -KO mice (Fig. S2D).

**Fig. 4:**
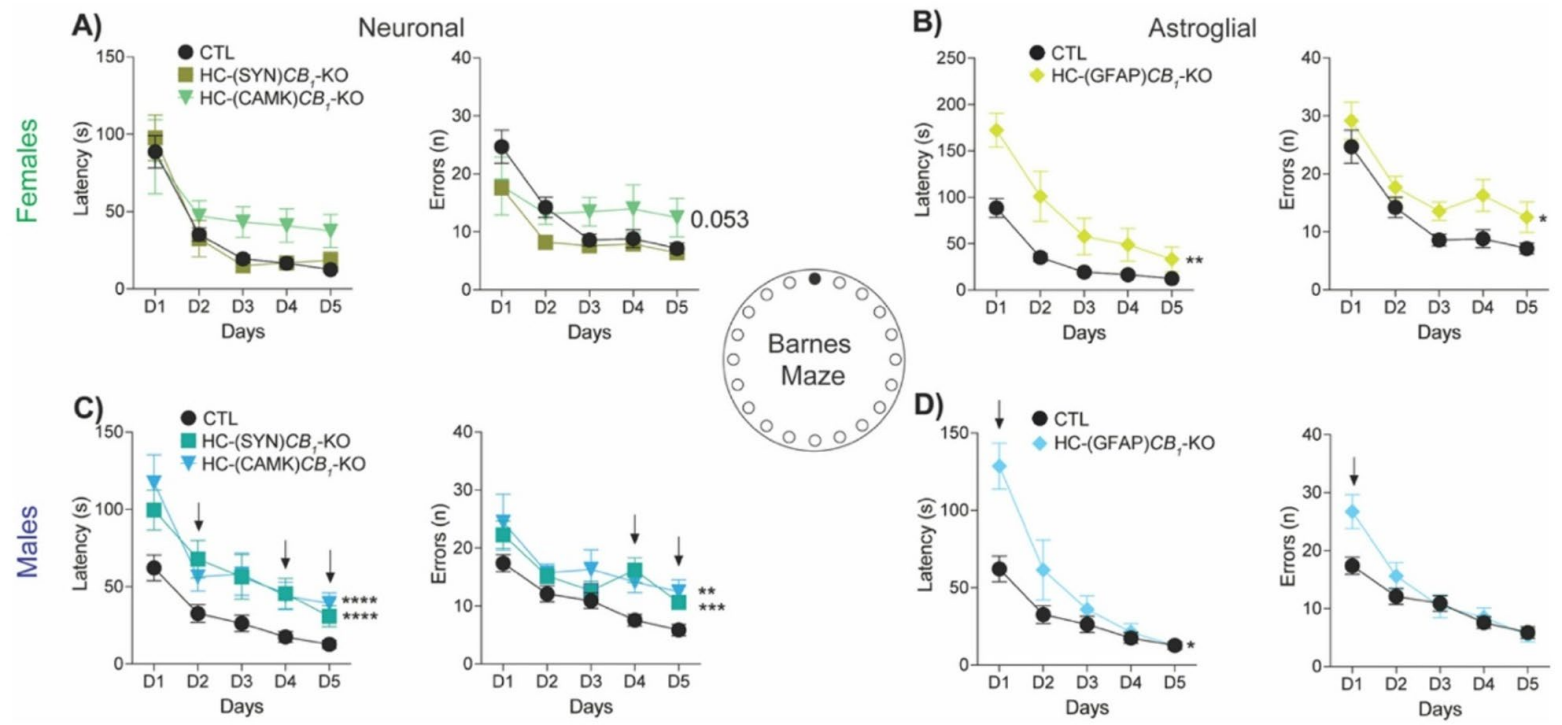
Learning and memory**.** Animals performed the Barnes maze for five consecutive days, and the latency and number of errors made before reaching the target hole were recorded. In females, neuronal deletion showed no alterations, except for an increased number of errors observed in the HC(CAMK)*CB*_*1*_-KO group **(A**,** right panel).** Females **(A**,** B)** with the astrocytic CB1 deletion showed a clear learning impairment, needing more time to reach the target hole **(B**,** left panel)**. The number of errors was also increased **(B**,** right panel)**. By contrast, significant differences were found in males **(C**,** D)** with CB1 deletion in neurons (HC(SYN)*CB*_*1*_-KO) and principal neurons (HC(CAMK)*CB*_*1*_-KO), increasing both the latency to reach the target hole **(C**,** left panel)** and the number of errors made **(C**,** right panel)**. HC(GFAP)*CB*_*1*_-KO animals also showed higher latency times **(D)**. Day differences were observed with the control ***(black arrow)***. Data are presented as mean ± S.E.M. Statistical analysis by Two-Way ANOVA test followed by Sidak’s multiple comparison test. * *p* < 0.05, ** *p* < 0.01, *** *p* < 0.001, *** *p* < 0.0001 vs. CTL. See Table S1 for detailed statistics

In the BM, mice learn the location of the hidden escape box using different visual cues in the behavioral room, and the test is repeated for 5 days. Hippocampal CB1 deletion produced a significant impairment in memory processes in both sexes (Fig. 4). Female HC-(GFAP)*CB*_*1*_-KO mice showed an increase in the latency to reach the target hole, along with an increase in the number of errors (Fig. 4B), compared to control mice. In male mice, CB1 deletion from neurons or astrocytes produced an increase in the latency to find the target hole (Fig. 4C and D) and we also observed an augmented number of errors in the neuronal groups (Fig. 4C). Global deletion of CB1 also affected the learning curve in the Barnes maze in females and males, with an increase in latency and no changes in the number of errors across trials in males (Fig. S2E and F).

Overall, we concluded that CB1 deletion from all neurons, particularly CAMKII-positive cells, in the HC of males negatively impacted both recognition and memory in the BM. By contrast, females’ memory processes were mainly impaired when CB1 was deleted from astrocytes.

### Navigation strategies

**Fig. 5:**
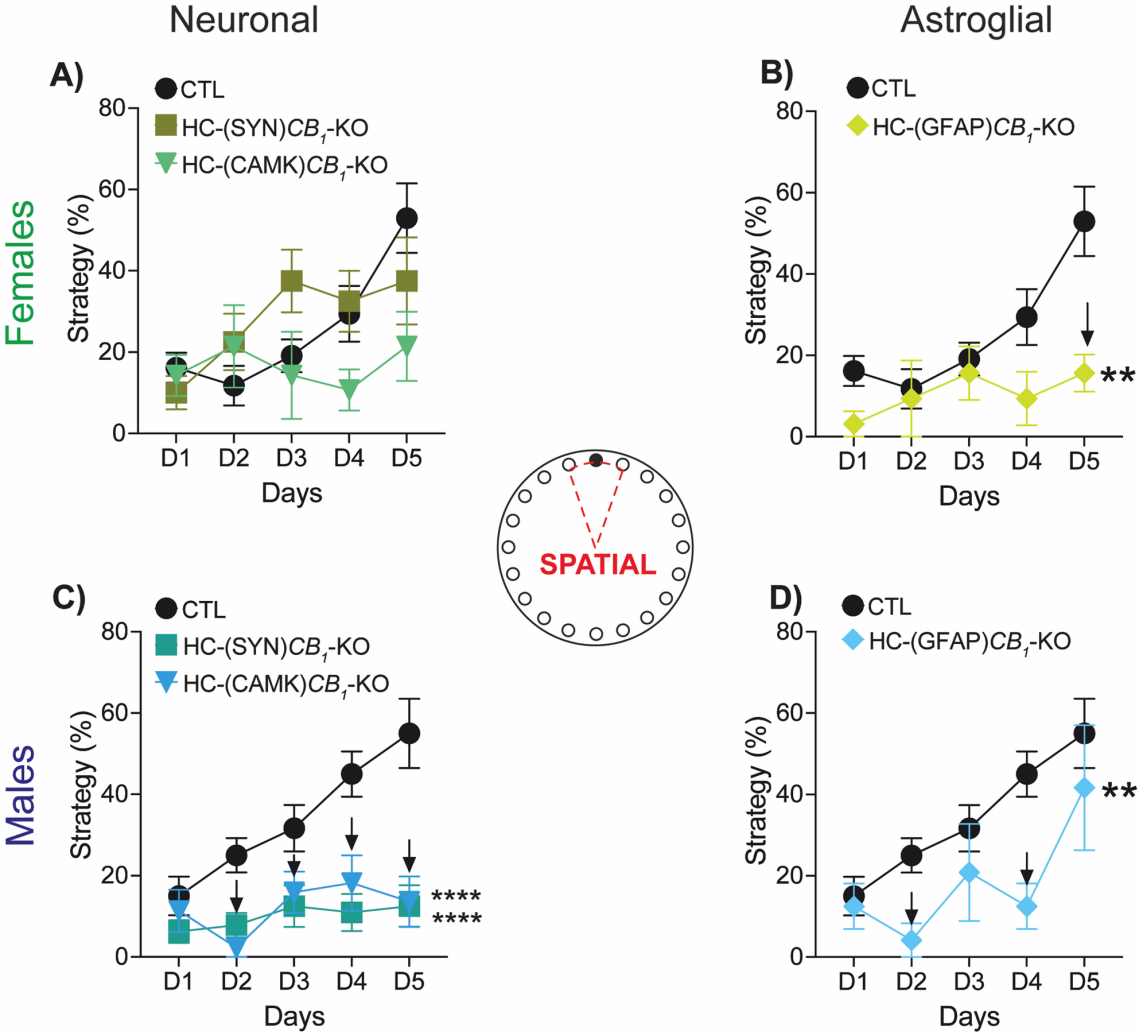
Navigation strategies. Over the test days, animals develop a Spatial strategy in which they go directly to the target hole using the visual cues. Astrocytic CB1 deletion in females **(A**,** B)** impaired the development of the Spatial strategy, which was significantly lower than that of CTL mice **(B)**. In males **(C**,** D)**, such impairment was also produced by neuronal **(C)** and astrocytic **(D)** CB1 deletion. Data are presented as mean ± S.E.M. Statistical analysis by Two-Way ANOVA test followed by Sidak’s multiple comparison test. ** *p*<0.01, **** *p*<0.0001 vs. CTL. *Black arrows* indicate *p*<0.05 vs. CTL on that specific day of the test. See Table S1 for detailed statistics

Animals use a wide range of navigation strategies to reach the escape box. In this work, we evaluated three different strategies, Spatial, Serial, and Random; with the Spatial strategy being more related to the hippocampal circuitry. In this regard, we found a significant effect on the overall use of Spatial strategies in both sexes but with a clear sexual dimorphism (Fig. 5). In male mice, CB1 deletion from pan-neuronal populations and principal neurons completely blunted the development and consolidation of the spatial strategy during the five days of the protocol (Fig. 5C). However, the impairment in HC-(GFAP)*CB*_*1*_-KO mice is milder and follows the same trend as controls (Fig. 5D). In female mice, the use of Spatial strategy was only significantly reduced in those mice where CB1 was deleted from astrocytes (Fig. 5A and B). These results are consistent with the animals’ overall performance in the BM, as assessed by latency and the number of errors committed before reaching the target hole.

Regarding the other strategies, the use of the Serial did not differ between males and females, regardless of the cell CB1 deletion (Fig. S3A-B). By contrast, the use of the Random strategy increased in both sexes (Fig. S3C-D), but with a distinct sexual dimorphism pattern. Males had a higher percentage of Random strategy after deleting CB1 from the global and principal neuron populations (Fig. S3D, left panel) and from astrocytes (Fig. S3D, right panel). Same results were obtained when the male *CB*_*1*_-KO group was characterized (Fig. S2I, right panel).

The use of Random strategy in female mice was increased in the HC-(GFAP)*CB*_*1*_-KO group (Fig. S3C, right panel), in line with the use of the Spatial strategy. Interestingly, HC-(CAMK)*CB*_*1*_-KO also showed an increased preference for the Random strategy (Fig. S3C, left panel), despite not affecting latency or the number of errors during the tests. Full *CB*_*1*_-KO females and males displayed a lower Serial strategy, and an increase in the Random one (Fig. S2H-I).

In summary, neuronal CB1 deletion in male mice and astrocytic deletion in females negatively impacted the use of Spatial strategy with a concomitant increase of the Random one.

### Nest building

**Fig. 6:**
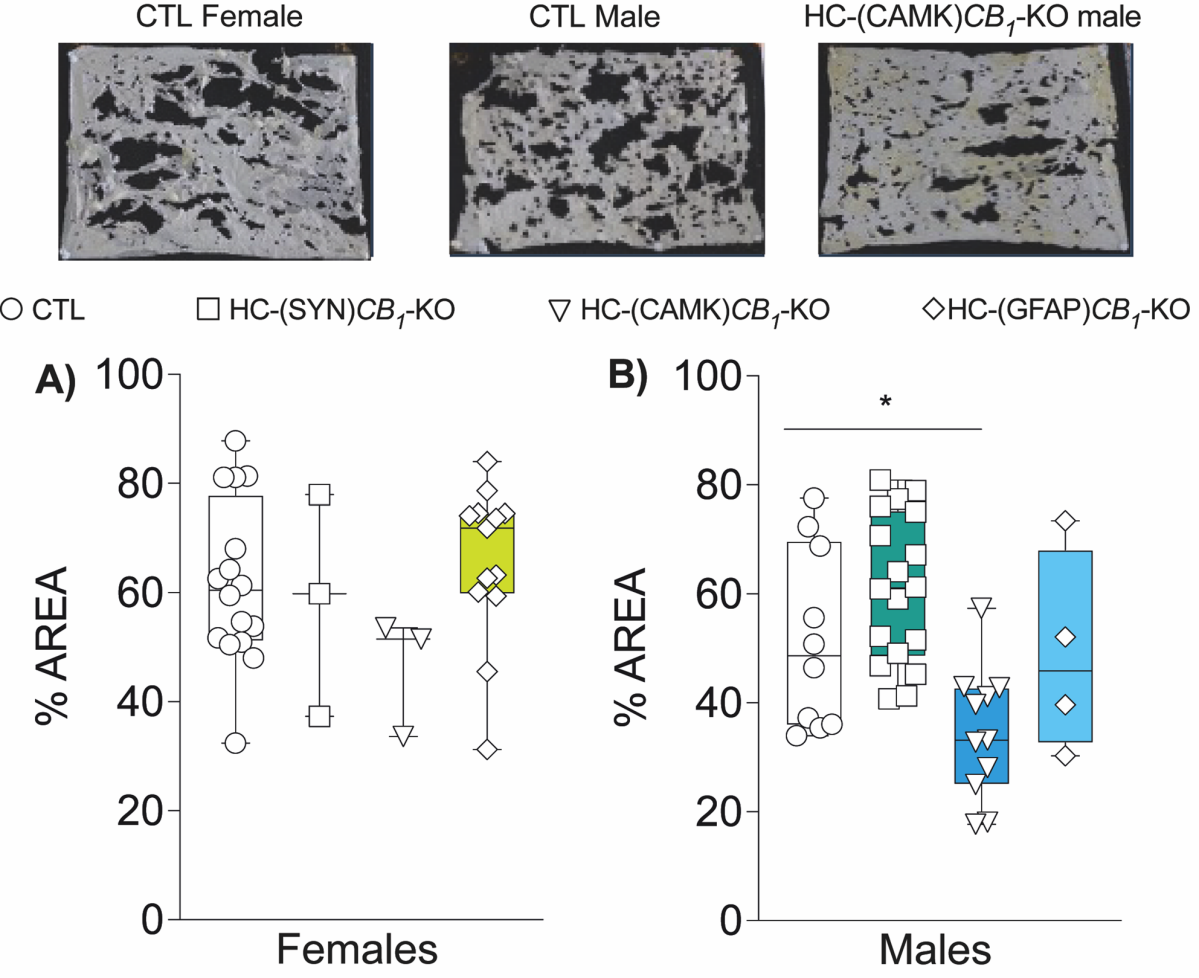
Nest building. Analysis of 2-week-old nests showed that **(A)** CB1 deletion in females did not affect paper nest working compared to controls. **(B)** Nest analysis in male animals showed significant differences for HC-(CAMK)*CB*_*1*_-KO group, with a lower percentage of intact remaining nest paper compared to all other neuronal groups and controls. Data are presented as mean ± S.E.M. Statistical analysis by One-Way ANOVA test followed by Sidak’s multiple comparison test. *p<0.05 vs. CTL. See Table S1 for detailed statistics

The nest building was analyzed one month after the surgery to study whether CB1 deletion could impact other complex hippocampal-dependent tasks. Consistent with the previous results obtained, only males were affected, while females’ nesting constructions were similar among groups (Fig. 6). HC-(CAMK)*CB*_*1*_-KO males showed an increased level of paper buildout compared to controls, with no difference observed in the astroglial or full neuronal deletion groups (Fig. 6B).

### Synaptic plasticity

**Fig. 7:**
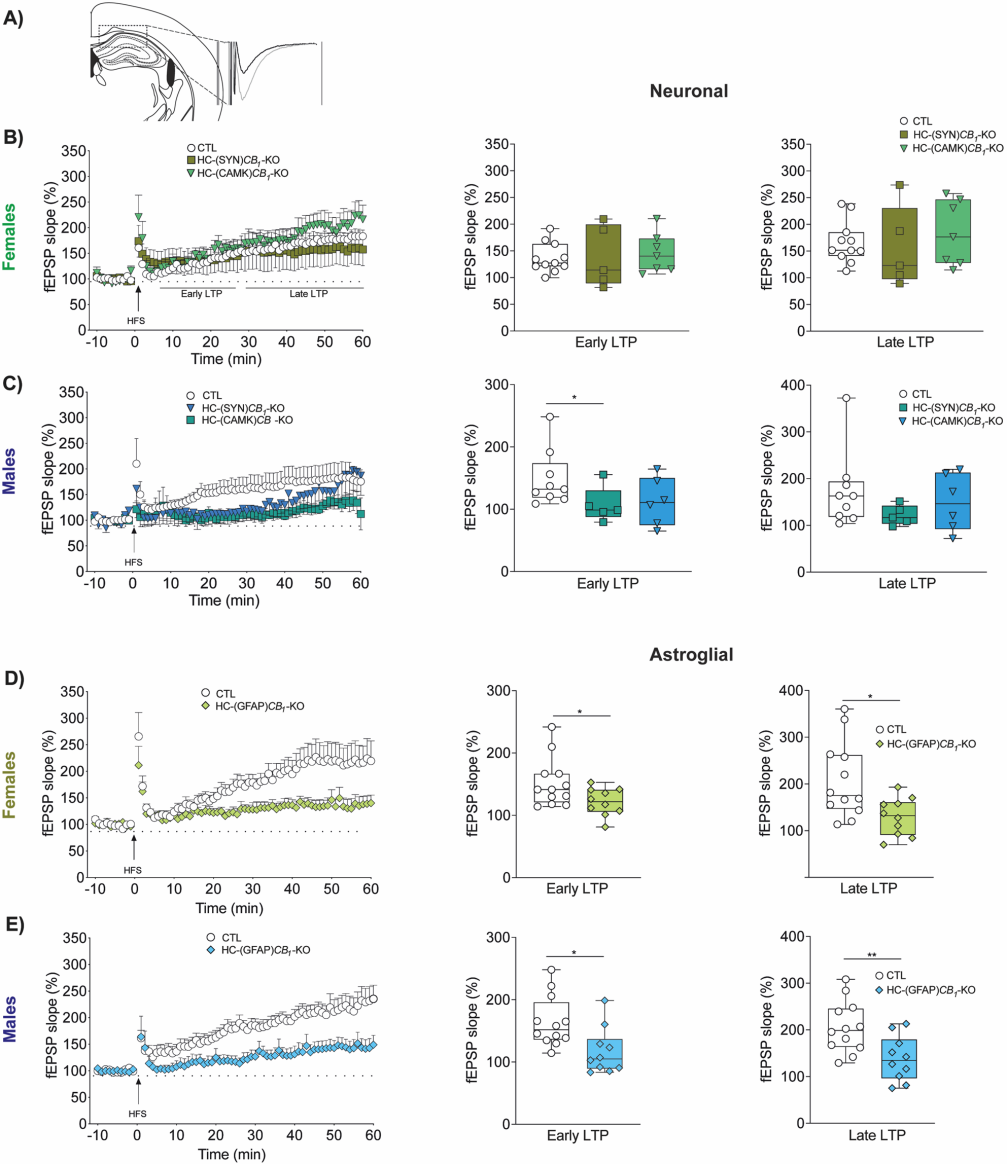
Synaptic plasticity is affected in males and females depending on CB1- cellular deletion. Field excitatory postsynaptic potential responses (fEPSPs) were measured in CA1 synapses after an HFS protocol was applied in the CA3 subfield of the hippocampus **(A)**. CB1 deletion in neurons reduced early-LTP induction only in males (**B and C**). By contrast, CB1 deletion in GFAP astrocytes (HC-(GFAP)*CB*_*1*_-KO) induced an LTP impairment in both female **(D)** and male animals **(E).** Data are presented as mean ± S.E.M. Statistical analysis by two-tailed unpaired Student *t* test or non-parametric Mann Whitney test. *p<0.05; **p<0.01. The number of experiments is indicated in parentheses as (slices tested/number of animals). See Table S1 for detailed statistics

To assess the possible mechanism of the impact on memory formation and navigation induced by cellular CB1 deletion, we measured synaptic alteration in the dorsal hippocampus by “ex vivo” electrophysiology (Fig. 7A). Field excitatory postsynaptic potential responses (fEPSPs) were measured in CA1 synapses after an HFS protocol was applied in the CA3 subfield of the hippocampus.

In female mice, neuronal CB1 deletion did not have a significant impact on the LTP induction (early LTP) or in the maintenance (late LTP) (Fig. 7B). Nevertheless, LTP development in males was significantly altered compared with controls, with a delayed induction of early LTP in HC-(SYN)*CB*_*1*_-KO mice (Fig. 7C). Interestingly, both LTP induction and maintenance were significantly impaired in those male and female mice in which CB1 was deleted in astrocytes (HC-(GFAP)*CB*_*1*_-KO) (Fig. 7D-E).

## Discussion

Previous studies have shown that CB1 signaling regulates referential and spatial memory [22, 38–40]. However, the specific cell types involved and sex-dependent mechanisms responsible for these functions remain unknown. The current work aims to elucidate the role of hippocampal CB1 receptors in memory and navigation. Through this study, we observed that male mice were consistently more affected by CB1 deletion in neurons. At the same time, females exhibited a milder phenotype, primarily linked to astroglial deletion.

The innate emotional behavior analysis showed a sexual dimorphism. Astrocytic CB1 deletion in females induced changes specifically in the elevated plus maze, a gold standard for the study of anxiety-like behaviors. On the other hand, male HC-(CAMK)*CB*_*1*_-KO mice exhibited altered emotional responses across the three tests. These animals had fewer entries into the center of the OF and the EPM, and longer times in the light zone of the light-dark box, which may be related to increased immobility or freezing. These results are aligned with recent studies that point out the role of CAMKII neurons of the hippocampus in anxiety and fear modulation, with their inhibition leading to attenuated anxiety-like behavior [41, 42]. Besides this neuronal role, hippocampal astrocytes also respond to anxiogenic environments, and optogenetic activation similarly reduces anxiety [43]. In this regard, HC-(GFAP)*CB*_*1*_-KO male mice displayed a reduced number of visits to the aversive zones in the three different tests, with a significant reduction of entries into the open arms of the elevated plus maze.

Our results show that neuronal CB1 receptors are necessary for spatial memory in male animals, whereas their deletion had no effect in females. By contrast, HC-(GFAP)*CB*_*1*_ deletion increased latency and the number of errors in both male and female mice, with a greater effect in the latter. Navigation is a complex task that requires the animal to identify and learn the objective position, and then navigate toward it using internal (egocentric) or external (allocentric) cues. BM results showed that spatial navigation was significantly reduced in males after deletion of CB1 from hippocampal neurons, either globally or specifically in principal neurons. This spatial memory impairment might also be related to hippocampal LTP, because only HC-(SYN)*CB*_*1*_-KO males, but not females, showed lower fEPSP slope. This could be partly explained by the fact that female mice do not use this strategy as often as males do. In our study, control females used the Serial strategy more than the Spatial strategy, potentially related to striatal function [5].

The CB1’s role in hippocampal information processing has been studied using various methodological approaches, including pharmacological and genetic approaches. Interestingly, both the agonism and antagonism of CB1 impair spatial memory [40, 44]. By contrast, this kind of memory was improved with low doses of THC in aged mice [39]. DAGL-KO mice (with lower 2-AG concentrations) exhibited a strong preference for non-Spatial strategies to solve the MWM [45]. In opposition, other researchers found no spatial memory impairment or alteration in navigation after pharmacologically increasing 2-AG or AEA levels before the BM test [46]. Another possible underlying mechanism is the participation of non-classical cannabinoid receptors. For instance, the use of the Serial strategy increased after administering a GPR55 agonist directly into male rats’ hippocampus, whereas an antagonist increased the use of the Random strategy [47].

CB1 expression in hippocampal glutamatergic neurons was also necessary for other hippocampal-related tasks, such as nest construction. Nest building is widespread across the animal kingdom and serves multiple functions, including reproduction, predator protection, hibernation, and thermoregulation, particularly in small rodents. Both male and female mice build nests of similar size, but female nest construction is influenced by hormonal and reproductive status, and nesting ability influences reproductive success [37]. Animals with hippocampal lesions show impaired nesting behavior, and the extent of hippocampal damage correlates with the severity of nest-building impairment [48], which is also seen in hippocampal-related diseases like Alzheimer’s Disease [49]. However, the cells and mechanisms behind this key hippocampal function remain unknown. Our findings show that hippocampal glutamatergic neurons’ CB1 is involved in nest building, but only in males. To our knowledge, this is the first report of such findings, and further research is needed to explore how hippocampal astrocytes contribute to this complex function.

Astrocytic CB1 receptors in the HC are necessary for learning processes and recognition memory in both male and female mice, as evidenced by the D.I. of the NOR test and by increases in latency and errors in the BM test mainly present in females. This recognition memory impairment was also described in full GFAP-*CB*_*1*_-KO mice [22] and the participation of astrocytic CB1 within the HC has also been described in working memory [38]. Further studies are needed to discern the underlying mechanism. One hypothesis involved the CB1’s regulation of neuronal NMDAR activity via D-serine availability, thereby modifying LTP [22]. In agreement with this, our results showed that the sole absence of astroglial CB1 in HC was sufficient to induce a decrease in LTP in both sexes, correlating with reference memory impairment. Besides this mechanism, astrocytic CB1 activation also upregulates glutamatergic transmission by releasing gliotransmitters, such as eCBs, which participate in the tripartite synapse [50]. The fact that GFAP is also expressed in hippocampal progenitor cells should also be considered. Astrocytic CB1 promotes astrocyte differentiation through endocannabinoid signaling [51]. Its manipulation may alter astrocyte-driven functions [52], potentially affecting neurogenesis, learning, and memory formation [53].

Concerning the potential influence of sex on the divergence observed in the early phase of the LTP induction following CB1 deletion in neuronal populations, it is notable that early LTP is impaired only in mice lacking CB1 in all neurons. However, it is preserved when CB1 is absent, specifically from glutamatergic cells. Multiple mechanisms through which CB1 contributes to distinct forms of synaptic plasticity have been previously described, depending on the cell type and recording site [54–56]. Among these, the interaction between CB1 and AMPARs may be particularly relevant in this context. AMPARs participate in neuronal depolarization and consequent intracellular calcium increase needed for the LTP maintenance, and its phosphorylation is regulated partially by CAMKII activity [57].

It is worth noting that CB1 receptor density in the brain differs significantly between sexes. Numerous studies have reported that males exhibit higher CB1 receptor density across most brain regions, whereas females exhibit an enhanced receptor response to agonist stimulation [58, 59]. This sexual dysmorphism has also been described in pathological conditions. Early-life exposure to ketamine impairs spatial memory retrieval in adult male but not female mice, probably due to a decrease of AKTmTOR pathway [60]. Chronic alcohol treatment in adult male mice shifted their learning from hippocampal-dependent pathways to striatum-dependent ones, which correlated to a decreased use of spatial memory [61]. In this line, a model of alcohol binge-drinking in young mice reduced CB1 density in excitatory terminals and astrocytes in the hippocampal formation of male animals, suggesting that this shift from one specific pathway to the other may be, at least in part, due to reduced CB1 density [62, 63].

## Conclusion

This study highlights the cell-type and sex-dependent modulation of CB1 receptors in hippocampal processes, specifically on recognition memory and navigation. Using CB1-flox transgenic mice in combination with viral vectors, we demonstrate that astrocytic CB1 receptors contribute to general learning and memory processes and associated CA1 synaptic plasticity in both sexes and may be involved in emotional behavior. In contrast, neuronal CB1, including CAMKII-expressing neurons, drives emotional behavior, spatial navigation, and synaptic plasticity mainly in males. These results reveal a pronounced sexual dimorphism in endocannabinoid signaling within the hippocampus, offering new insight into how cell type and sex jointly shape the neural substrates of memory, navigation, and emotion. Future research will explore the contributions of other cell types, such as D1 + cells, as well as the subcellular localization of CB1 and its potential sex-specific differences.

## Electronic Supplementary Material

Below is the link to the electronic supplementary material.

Supplementary Material 1

## Data Availability

The datasets used and/or analyzed during the current study are available from the corresponding author on reasonable request.
