# Supplementary material for: Neuroglial CB1 receptors modulate hippocampal processes in a sex-dependent manner
Source: Biol Sex Differ. 2026 Mar 19;17:102. doi: 10.1186/s13293-026-00886-w (PMC13162385; doi:10.1186/s13293-026-00886-w)
Supplement: Supplementary file 1 — Supplementary Material 1 [file 13293_2026_886_MOESM1_ESM.docx]

| **Supplementary Table 1. Baseline participant characteristics according to quartile for serum sex hormone-binding globulin (SHBG) in men and women** | | | | | | | | |
| --- | --- | --- | --- | --- | --- | --- | --- | --- |
|  | **Quartile for SHBG in men (n = 176,909)** | | | | **Quartile for SHBG in women (n = 160,147)** | | | |
|  | **Q1** | **Q2** | **Q3** | **Q4** | **Q1** | **Q2** | **Q3** | **Q4** |
| Age, y | 53 (46-60) | 57 (49-62) | 59 (51-64) | 61 (55-65) | 57 (50-62) | 57 (50-63) | 56 (49-62) | 54 (47-62) |
| Ethnicity, % |  |  |  |  |  |  |  |  |
| White | 93.1 | 95.3 | 96.3 | 97.2 | 93.7 | 95.5 | 96.5 | 96.9 |
| Asian or Asian British | 4.4 | 2.4 | 1.8 | 1.3 | 3.3 | 1.8 | 1.2 | 0.9 |
| Black or Black British | 1.8 | 1.7 | 1.5 | 1.1 | 2.2 | 2.0 | 1.7 | 1.5 |
| Mixed | 0.7 | 0.5 | 0.5 | 0.4 | 0.7 | 0.7 | 0.7 | 0.7 |
| TDI | -2.0 (-3.6-0.9) | -2.2 (-3.7-0.5) | -2.2 (-3.7-0.4) | -2.2 (-3.7-0.6) | -2.0 (-3.5-0.8) | -2.2 (-3.7-0.4) | -2.3 (-3.7-0.2) | -2.2 (-3.7-0.3) |
| Smoking status, % |  |  |  |  |  |  |  |  |
| Never | 51.8 | 50.3 | 48.9 | 47.4 | 60.6 | 60.4 | 61.1 | 61.8 |
| Former | 37.8 | 38.4 | 38.4 | 37.0 | 31.4 | 30.7 | 30.0 | 28.7 |
| Current | 10.4 | 11.3 | 12.6 | 15.6 | 8.0 | 8.9 | 9.3 | 9.5 |
| Drinking status, % |  |  |  |  |  |  |  |  |
| Never | 3.4 | 2.6 | 2.4 | 2.7 | 7.3 | 5.4 | 4.9 | 5.1 |
| Former | 3.0 | 2.9 | 3.2 | 4.2 | 3.5 | 2.9 | 2.8 | 3.6 |
| Current | 93.6 | 94.5 | 94.5 | 93.1 | 89.3 | 91.7 | 92.3 | 91.3 |
| Total PA, MET-h/week | 25.6 (11.0-53.8) | 29.3 (12.9-59.6) | 32.4 (14.9-64.9) | 35.6 (16.6-71.1) | 24.4 (11.0-50.9) | 28.8 (12.9-54.7) | 30.4 (14.8-58.2) | 32.4 (15.6-60.9) |
| Coffee, cup/day | 2 (0.5-3) | 2 (0.5-3) | 2 (0.5-3) | 2 (0.5-3) | 1 (0-3) | 1 (0.5-3) | 1 (0.5-3) | 1 (0.5-3) |
| Tea, cup/day | 3 (1-5) | 3 (1-5) | 3 (1-5) | 3 (-5) | 3 (1-5) | 3 (1-5) | 3 (1-5) | 3 (1-5) |
| Diabetes, % | 9.8 | 6.7 | 5.6 | 4.9 | 7.6 | 2.8 | 1.7 | 1.6 |
| HbA1c, mmol/mol | 35.8 (33.1-39.1) | 35.3 (32.8-38.1) | 35.1 (32.6-37.7) | 34.9 (32.5-37.4) | 36.6 (34.0-39.5) | 35.2 (32.9-37.6) | 34.6 (32.3-36.9) | 33.9 (31.6-36.1) |
| Hypertension, % | 33.7 | 32.2 | 31.6 | 29.2 | 33.0 | 23.7 | 18.2 | 14.1 |
| SBP, mmHg | 141 (130-153) | 141 (130-154) | 141 (129-154) | 141 (129-154) | 139 (127-153) | 136 (123-151) | 133 (121-148) | 130 (118-145) |
| Hyperlipidemia, % | 26.2 | 24.3 | 23.5 | 21.3 | 20.6 | 12.9 | 9.6 | 6.7 |
| Total cholesterol, mmol/L | 5.5 (4.8-6.3) | 5.5 (4.7-6.3) | 5.5 (4.7-6.2) | 5.4 (4.7-6.2) | 5.8 (5.1-6.7) | 5.9 (5.1-6.6) | 5.8 (5.1-6.6) | 5.7 (5.1-6.5) |
| Aspirin, % | 17.6 | 18.2 | 18.5 | 18.5 | 12.5 | 9.1 | 7.6 | 6.7 |
| NSAID, % | 14.6 | 13.3 | 11.9 | 10.6 | 18.7 | 17.5 | 16.6 | 16.6 |
| C-reactive protein, mg/L | 1.6 (0.8-3.0) | 1.3 (0.7-2.6) | 1.2 (0.6-2.3) | 1.0 (0.5-2.1) | 2.3 (1.2-4.6) | 1.5 (0.7-2.9) | 1.0 (0.5-2.1) | 0.8 (0.4-1.7) |
| Body mass index, kg/m^2^ | 28.8 (26.5-31.6) | 27.7 (25.5-30.3) | 26.9 (24.7-29.4) | 25.8 (23.6-28.3) | 29.5 (26.4-33.4) | 26.8 (24.3-30.0) | 25.1 (23.0-27.9) | 23.8 (21.9-26.2) |
| Waist circumference, cm | 99 (93-107) | 97 (91-104) | 95 (89-102) | 92 (85-99) | 92 (84-101) | 85 (78-93) | 80 (74-87) | 76 (71-83) |
| Being menopausal, % | NA | NA | NA | NA | 74.2 | 71.4 | 67.4 | 59.7 |
| Ever used HRT, % | NA | NA | NA | NA | 35.2 | 34.5 | 33.2 | 33.1 |
| HRT, hormone replacement therapy; MET, metabolic equivalent; NA, not applicable; NSAID, non-steroidal anti-inflammatory drugs; PA, physical activity; TDI, Townsend deprivation index.  Data are presented as median (IQR) for continuous variables and as percentage for categorical variables. | | | | | | | | |

**Supplementary Table 2. Sensitivity analysis of the association between calculated free testosterone and risk of cholecystectomy in men and women.**

| **Sex-specific quartiles for cFT** | **Men HR (95% CI)** | **Women HR (95% CI)** |
| --- | --- | --- |
| Q1 | 1.00 (referent) | 1.00 (referent) |
| Q2 | 1.02 (0.92–1.12) | 1.25 (1.14-1.37) |
| Q3 | 0.98 (0.88–1.09) | 1.48 (1.35–1.61) |
| Q4 | 1.00 (0.90–1.12) | 2.03 (1.87–2.21) |
| **P-trend** | 0.857 | <0.001 |
| **Per SD increment** | 1.00 (0.96–1.04) | 1.25 (1.22–1.28) |

Calculated free testosterone (cFT) was derived using the Vermeulen mass action equation based on measured total testosterone, sex hormone–binding globulin (SHBG), and serum albumin.

Models were adjusted for age (y), ethnic group (White, Asian/Asian British, Black/Black British, mixed), Townsend deprivation index, smoking (never, former, current [<10, 10-<50, ≥50 pack-years]), alcohol drinking (never, former, current<10 g/d in men or <5 g/d in women, current 10-<25 g/d in men or 5-<15 g/d in women, current ≥25 g/d in men or ≥15 g/d in women), total physical activity (MET-h/week), and for women, menopausal status (yes, no) and hormone replacement therapy (ever, never).

**Supplementary Figure 1. Flow chart of participant selection.**

**Supplementary Figure 2. Subgroup analysis for the association of serum SHBG (per 1-SD increment) with risk of cholecystectomy in men and women.**

Results were adjusted for the covariates listed for model 3 of Table 1 in the article.

HRT, hormone replacement therapy; NA, not applicable.


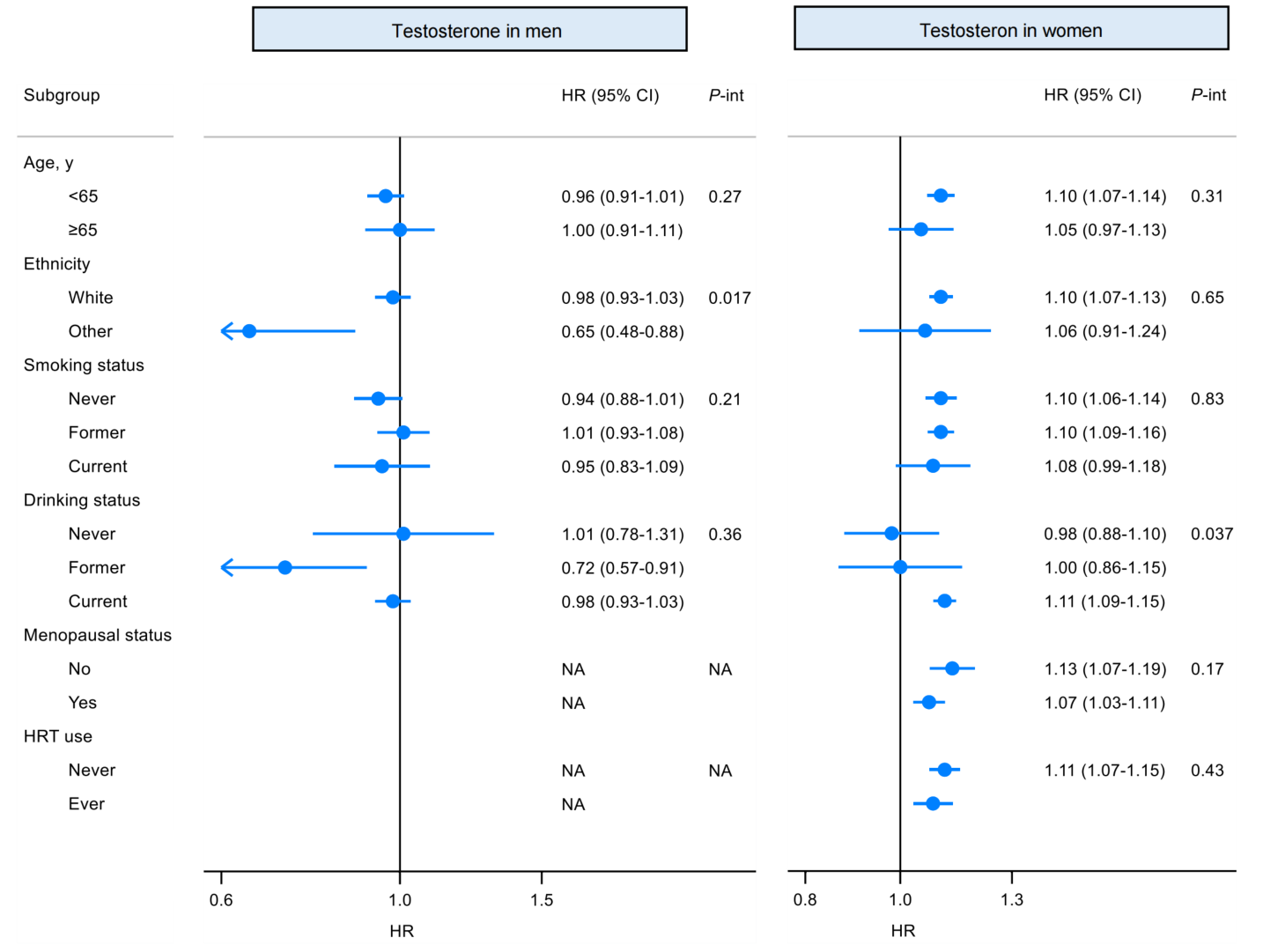


**Supplementary Figure 3. Subgroup analysis for the association of serum testosterone (per 1-SD increment) with risk of cholecystectomy in men and women.**

Results were adjusted for the covariates listed for model 3 of Table 2 in the article.

HRT, hormone replacement therapy; NA, not applicable.

**Supplementary Figure 4. Sensitivity analysis for the association of serum SHBG (per 1-SD increment) with risk of cholecystectomy in men and women.**

Covariates for model 3 are listed in the footnote for Table 1 in the article.

Excluding A: excluding 28,382 men (671 cholecystectomies) and 8181 women (366 cholecystectomies) who had non-cancer diseases of the reproductive systems at baseline. For men, these included diseases affecting the prostate, testis, and epididymis (ICD-10 codes: N40-N42, N44-N45); for women these included ovarian diseases (ICD-10 codes: N83, E28).

Excluding B: excluding 6634 men (151 cholecystectomies) and 19,916 women (822 cholecystectomies) who had thyroid disorders at baseline defined by ICD-10 codes: E01-E07.

Excluding C: excluding 346 men (of the 2877 cholecystectomies) and 420 women (of the 4607 cholecystectomies) who were not diagnosed with cholelithiasis (ICD-10 codes K80) before cholecystectomies were performed.

BMI, body mass index; CRP, C-reactive protein; HbA1c, glycosylated hemoglobin; NSAID, non-steroidal anti-inflammatory drugs; SBP, systolic blood pressure; SHBG, sex hormone-binding globulin; TC, total cholesterol; WC, waist circumference.

**Supplementary Figure 5. Sensitivity analysis for the association of serum testosterone (per 1-SD increment) with risk of** **cholecystectomy in men and women.**

Covariates for model 3 are listed in the footnote for Table 2 in the article.

Excluding A: excluding 28,382 men (671 cholecystectomies) and 8181 women (366 cholecystectomies) who had non-cancer diseases of the reproductive systems at baseline. For men, these included diseases affecting the prostate, testis, and epididymis (ICD-10 codes: N40-N42, N44-N45); for women these included ovarian diseases (ICD-10 codes: N83, E28)

Excluding B: excluding 6634 men (151 cholecystectomies) and 19,916 women (822 cholecystectomies) who had thyroids disorder at baseline defined by ICD-10 codes: E01-E07.

Excluding C: excluding 346 men (of the 2877 cholecystectomies) and 420 women (of the 4607 cholecystectomies) who were not diagnosed with cholelithiasis (ICD-10 codes K80) before cholecystectomies were performed.

BMI, body mass index; CRP, C-reactive protein; HbA1c, glycosylated hemoglobin; NSAID, non-steroidal anti-inflammatory drugs; SBP, systolic blood pressure; TC, total cholesterol; WC, waist circumference.
